# Applications of CRISPR-Cas9 in Alzheimer’s Disease and Related Disorders

**DOI:** 10.3390/ijms23158714

**Published:** 2022-08-05

**Authors:** Laura M. De Plano, Giovanna Calabrese, Sabrina Conoci, Salvatore P. P. Guglielmino, Salvatore Oddo, Antonella Caccamo

**Affiliations:** 1 Department of Chemical, Biological, Pharmaceutical and Environmental Sciences, University of Messina, Viale Ferdinando Stagno d’Alcontres, 31, 98168 Messina, Italy; 2 Department of Drug and Health Sciences, University of Catania, Viale Andrea Doria 6, 95125 Catania, Italy

**Keywords:** Huntington’s disease, Parkinson’s disease, amyotrophic lateral sclerosis, transgenic mice, gene editing, neurodegeneration, brain

## Abstract

Alzheimer’s disease, Parkinson’s disease, amyotrophic lateral sclerosis, and Huntington’s disease represent some of the most prevalent neurodegenerative disorders afflicting millions of people worldwide. Unfortunately, there is a lack of efficacious treatments to cure or stop the progression of these disorders. While the causes of such a lack of therapies can be attributed to various reasons, the disappointing results of recent clinical trials suggest the need for novel and innovative approaches. Since its discovery, there has been a growing excitement around the potential for CRISPR-Cas9 mediated gene editing to identify novel mechanistic insights into disease pathogenesis and to mediate accurate gene therapy. To this end, the literature is rich with experiments aimed at generating novel models of these disorders and offering proof-of-concept studies in preclinical animal models validating the great potential and versatility of this gene-editing system. In this review, we provide an overview of how the CRISPR-Cas9 systems have been used in these neurodegenerative disorders.

## 1. Introduction

Genome editing is of great interest for the understanding and treatment of human diseases [1]. In this context, CRISPR-Cas9 systems represent a smart toolbox for a variety of therapeutic and biotechnology applications. It is a natural genome editing system that bacteria use as an immune defense [2]. When infected by viruses, bacteria capture small pieces of the viral genome and insert them into their own DNA, creating segments known as clustered regularly interspaced short palindromic repeats (CRISPR) loci; this process is known as spacer acquisition. The CRISPR loci are then transcribed, and the RNA is processed to generate crispr RNAs (cRNAs) that serve as “guides” for endogenous Cas9 enzymes; this process is known as the targeting phase. Should bacteria be reinfected by the same virus, the guide RNA will direct Cas9 to cleave the viral genome [2].

This immune defense system has been adapted to edit the genome of mammalian cells. The versatility of the CRISPR system is mainly due to the possibility of editing any nucleic acid sequence through the engineering of a short fragment of RNA, which can be easily designed to bind specific target sequences of the genome of the target cell. The guide RNA recognizes the intended DNA sequence through direct base pairing and redirects the Cas-endonuclease on it. Once the DNA has been cleaved, the double-strand break is repaired by homologous recombination (HR) or non-homologous end-joining (NHEJ). The former allows for an accurate correction of the damage, while the latter leads to the generation of indels. By combining HR with a customized sequence of DNA, one can add, replace, or delete even single nucleotides [2].

A modified Cas9 enzyme has been obtained by introducing two independent point mutations (D10A and H840A) into the wildtype sequence of the endonuclease domain of the enzyme. The mutant Cas9, known as dead Cas9 (dCas9), is capable of binging to the gRNAs and the target DNA but, having lost its endonuclease activity, cannot cut it. In other words, paired with the proper gRNA, dCas9 retains the selectivity of Cas9 without the ability to cut the target DNA [3]. dCas9 is normally used to induce transcriptional activation or repression of a target gene, which can be obtained by fusion of an activator or a repressor to the Cas9 protein, respectively. Similarly, one can fuse to Cas9 enzymes involved in epigenetic modification, thereby creating a molecular tool to selectively and precisely modify the epigenetic makeup of a gene [4]. Another commonly used variation of the traditional CRISPR-Cas9 system is known as Cas9 nickase (nCas9). This engineered nCas9 enzyme is obtained by introducing the D10A point mutation into the wildtype sequence of the protein [5]. In doing so, one catalytic domain of Cas9 is maintained while the other is removed. The end product is a modified enzyme that cleaves only a single filament of DNA [6]. Given that single-strand breaks are easily repaired by the cell, to obtain indels at the target site, it is necessary to use two different gRNAs, one per strand. By doing so, one can drastically reduce the probability of off-target genome modifications [7,8]. Both dCas9 and nCas9 have been successfully used in preclinical studies of neurodegenerative disorders (see below).

Overall, the CRISPR-Cas9 system, with its variations, is considered a great promise for the treatment of human genetic diseases, including cardiovascular diseases, neurodegenerative disorders, and cancer [9,10,11], and to generate novel animal models of Alzheimer’s disease (AD) and related disorders [6]. In this work, we will provide a short historical perspective of the CRISPR-Cas9 system and discuss its advantages and limitations. We will then focus on how this system has been used in various neurodegenerative disorders, which include AD, Parkinson’s disease (PD), amyotrophic lateral sclerosis (ALS), and Huntington’s disease (HD; Table 1).

## 2. Brief History of the CRISPR-Cas9 System

CRISPR is a fundamental part of a microbial adaptive immune system, which recognizes foreign nucleic acids based on their sequence and eliminates them by Cas9. The first description of CRISPR loci appeared in 1987 when repeats of 29 nucleotides were found downstream of the *iap* gene of Escherichia coli; these sequences were interspaced by non-repetitive ones of 32 nucleotides [12]. Over the following years, the sequencing of more microbial genomes highlighted the high frequency of these element repeats, later defined as CRISPR loci [13]. In 2002, it was reported that the CRISPR loci are transcribed into small noncoding RNAs (crRNA) containing one spacer sequence used as a guide, which serves to identify foreign nucleic acids and guide Cas9 to cleave them [14]. Subsequently, Haft and colleagues identified CRISPR-associated (Cas) genes as a basis for the eventual classification of three different types of CRISPR systems [15]. In the same year, a systematic analysis of the spacer sequences found between element repeats suggested their extrachromosomal and phage-associated origins [13,16,17]. These findings, together with previous studies showing that when CRISPR loci are transcribed, viruses are unable to infect archaeal cells, led to the speculation that CRISPR arrays operate as a defense mechanism against bacteriophage infection [13,18]. From that, several hypotheses were raised, including that CRISPR spacers act as small RNA guides to degrade viral transcripts in an RNAi-like mechanism [19] or that CRISPR spacers direct Cas enzymes to cleave viral DNA at spacer-matching regions [16]. Subsequent experiments highlighted the natural role of CRISPR-Cas in the adaptive immune system of bacteria and helped to establish the basic functions and mechanisms of three types of CRISPR loci [20].

Starting from 2010, scientists had begun to harness the natural CRISPR system for various biotechnological applications in several fields, including medicine, plant science, and animal breeding. Indeed, the CRISPR-Cas9 system has been adopted on human and mouse cell lines [5,21,22] and animal models of neurodegenerative diseases [23,24].

## 3. CRISPR Types and Molecular Mechanisms

**Figure 1:**
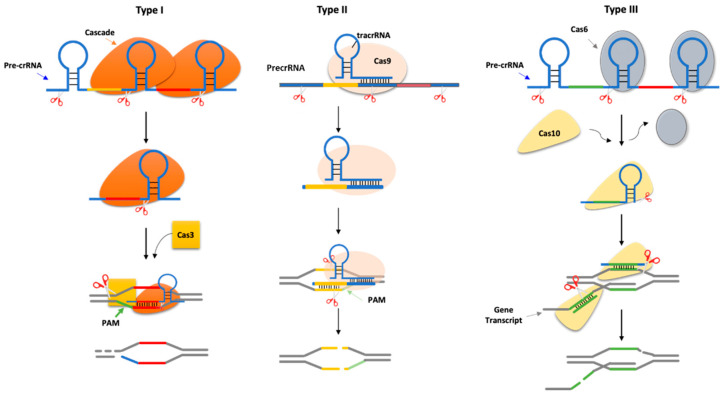
CRISPR-Cas types. Type I systems. Cascade (Cas protein complex) cleaves at the base of the stem-loop structure of each repeat in the long precursor crRNA (pre-crRNA), generating short crRNA guides. The complex matches with the target DNA (known as protospacer), which is flanked by a protospacer-adjacent motif (PAM). The annealing recruits Cas3 nuclease, which cleaves the DNA target downstream of the PAM sequence and degrades the opposite strand. Type II systems. These systems encode a small RNA known as trans-encoded crRNA (tracrRNA), which is bound by Cas9 and has regions of complementarity to the repeat sequences in the pre-crRNA. The complex is cleaved by RNase III to generate crRNA guides/Cas9 complex, which in turn cleaves both strands of the protospacer/crRNA R-loop. A PAM sequence is located downstream of the target sequence. Type III systems. Cas6 is a repeat-specific endoribonuclease that cleaves the pre-crRNA at the base of the stem-loop structure of each repeat. The crRNA is loaded into the Cas10 complex, where it is further trimmed at the 3′ end to generate a mature crRNA. The Cas10 requires DNA target transcription to cleave the non-template strand of the protospacer DNA and crRNA-guided transcript.

The CRISPR-Cas systems can be classified into three different types based on the accessory Cas gene family [25]. Although they have the same molecular mechanism dependence on crRNA, they differ in the biogenesis of crRNAs and the targeting requirements (Figure 1).

Type I CRISPR-Cas: immunity is mediated by the Cascade complex and the Cas3 nuclease [8]. Selective subunits of Cascade, known as Cas6, cleave the precursor crRNA producing short crRNAs that remain associated with Cascade and are used to locate a complementary sequence in the target DNA known as the protospacer [26,27,28]. Another subunit of Cascade, Cas8 (also known as CasA or Cse1), recognizes a short sequence motif located immediately upstream of the target sequence identified by the crRNA, named protospacer adjacent motif PAM [29]. PAM promotes the binding of Cascade to the target and the formation of the R-loop between the crRNA spacer sequences and the dsDNA. The first eight base pairs at the 5′ end of the crRNA-DNA duplex are critical for immunity and define a trigger sequence within the target [8].

Type II CRISPR-Cas: it does not require the Cascade system, but it utilizes only the Cas9 gene [30]. In addition, as opposed to the other CRISPR types, it needs two small RNAs: the crRNA and the trans-Cas system (tracrRNA) [31]. The tracrRNA forms a secondary structure that mediates the association with Cas9 and is complementary to the repeat sequences of the CRISPR array [31]. The dsRNA formed between the tracrRNA and the precursor crRNA is cleaved by RNase III, resulting in the cleavage of each repeat and the processing of the long CRISPR transcript into small crRNA guides [31]. PAM, which is located downstream of the target, is recognized by a PAM-binding domain present in Cas9. The transient binding of Cas9 to PAM sequences within the target DNA promotes the melting of the two DNA strands upstream of the PAM, between 6–8 bases of the spacer sequence of the crRNA guide, thereby triggering the formation of an R-loop and target cleavage [32,33].

Type III CRISPR-Cas: the precursor crRNA is cleaved at its 5′ by a repeat-specific endoribonuclease, Cas6. The small crRNAs generated after Cas6 cleavage become part of the Cas10–Csm or Cas10–Cmr complex for type III-A or III-B systems, respectively. Within these complexes, the crNRAs undergo further processing by cleavage of their 3′. Type III CRISPR-Cas does not require a PAM sequence [34,35].

## 4. Limitations

Compared to other editing techniques, the CRISPR-Cas9 system takes genome engineering to the next level of molecular engineering as it provides greater efficiency, feasibility, and multi-role clinical application [1]. However, the possibility of off-target cleavages by Cas9 may confound this technique’s application [36]. Indeed, the clinical utility of CRISPR-Cas gene therapy mainly depends on the accuracy of genetic changes and the precision of the interaction between gRNA/target DNA. However, inaccurate (off-target) genome editing could occur when CRISPR-induced DNA cleavage and repair happens at an unwanted genomic location. For example, different alleles of an edited gene are created when non-homologous end joining and/or homology-directed repair are performed by the cell. Moreover, after genome editing in mice, between 10 and 1000 off-targets binding sites of Cas9 were observed, e.g., [36,37]. These events, which occur in part because the gRNAs can tolerate several mismatches, represent significant disadvantages in a clinical setting, but they can be reduced using dedicated algorithms for the section of guide RNAs [38]. Although a scientific consortium has been instituted by the National Institute of Standards and Technology (NIST) to measure and standardize the results in the progression of the genome-editing method, the issues of delivery, potency, and specificity of CRISPR-Cas methods remain an area of active investigation.

## 5. CRISPR-Cas9 in Alzheimer’s Disease

AD, the most common neurodegenerative disorder, is characterized by the accumulation of tau and amyloid-β (Aβ). The former forms intracellular neurofibrillary tangles, while the latter forms extracellular amyloid plaques [39]. A proper diagnosis of AD is made after a postmortem evaluation of the brain, even though novel approaches for an easy, non-invasive diagnosis are being developed [40,41,42,43]. While the vast majority of AD cases are sporadic, a small percentage can be attributed to dominant, autosomal mutations in one of three genes, amyloid precursor protein (APP), presenilin- (PS) 1 and 2 [44].

In the past years, most of the animal models of AD were generated by overexpressing human mutated genes involved in the production of Aβ and tau. These models have proven to be extremely useful for understanding some aspects of AD pathogenesis, but their utility is limited because most of the AD mouse models do not develop frank neurodegeneration [45]. Recently, CRISPR-Cas9 technology has been used to create new AD models that show a more accurate disease phenotype, elucidate mechanisms of pathogenesis, screen pathogenic genes, and at the same time, find a therapy for this insidious disorder [46]. We, briefly, described below the most significant examples.

While most of the known mutations in the APP gene are causative of the disease, some mutations/variations are protective against AD [47,48]. Along these lines, Saido and colleagues identified protective deletions within the 3′-UTR of APP [49]. When these deletions were introduced using CRISPR-Cas9 within the APP of APP-KI mice (an animal model of AD), they significantly reduced Aβ pathology, which in these mice is driven by three human mutations within the mouse APP gene [49].

Bart De Strooper and his group used CRISPR-Cas9 to generate novel animal models of AD. Specifically, they converted the endogenous mouse and rat APP gene into the human version by introducing three independent point mutations, G676R, F681Y, and R684H. In doing so, they generated humanized animal models that will be useful for studying APP processing and unveiling new mechanisms of disease pathogenesis [50]. Tau knockout mice are useful to study the involvement of this protein in AD. Tan and colleagues used CRISPR-Cas9 to introduce a short deletion in the transcriptional start codon in exon 1 of the Mapt gene, which encodes tau. This new animal model, generated on a pure C57Bl/6J background, is resistant to excitotoxicity and does not show any memory deficit [51].

CRISPR-Cas9 has also been used to identify how known risk factors for AD contribute to disease pathogenesis. To this end, Plcγ2, a gene expressed in microglia, has been associated with AD. As for the APP gene, there are different variants of the Plcγ2 gene, some associated with a high risk of developing AD, while others are associated with a lower risk of developing the disease [52]. Among these, the Plcγ2-P522R variant reduces the risk of developing AD [53]. To identify the molecular link between Plcγ2 and AD, Christian Haass and colleagues generated a Plcγ2-P522R knock-in mouse model using CRISPR-Cas9 and found that this variant may reduce the risk of AD by increasing microglial function [54].

Several groups have shown the potential therapeutic approaches of the CRISPR-Cas9 system in AD. For example, György and colleagues using the Tg2576 mice, an animal model of AD expressing mutant human APP, showed that CRISPR-Cas9-mediated disruption of the APP mutant gene was sufficient to decrease AD-like pathology [54]. The validity of this approach has been recently confirmed [55]. However, as a therapeutic approach, the disruption of the mutant, disease-causing allele is limited to familial AD cases, as in sporadic cases, such alleles are not present. To bypass this potential limitation, the CRISPR-Cas9 system has been used to target the C-terminus of the endogenous APP gene and reduce Aβ production. Specifically, the authors set to shift the processing of APP away from the amyloidogenic β-cleavage of APP while up-regulating the neuroprotective APP-α-cleavage. They showed both in vitro and in vivo that their approach was efficacious at reducing Aβ production with minimal off-site effects [56].

Another strategy to reduce Aβ production sees the employment of Cas9 activators or inhibitors: in this case, a catalytically inactive Cas9 (known as dCas9) is combined with gene activators or inhibitors. Specifically, dCas9 is driven to the target gene by the guide RNA, but it will not introduce DSBs. Instead, it will carry with it a general transcriptional activator or inhibitor that may increase or decrease the expression of the target gene [57]. To facilitate the delivery of Cas9 activators, several groups have generated Cas9 activator nanocomplexes, non-viral delivery systems with excellent potential therapeutic applications, even for CNS diseases [58,59]. These approaches have been successfully used in vivo to decrease the expression of BACE-1 or increase the expression of ADAM10 in animal models of AD. In both cases, the result was the reduction of Aβ production and the concomitant amelioration of AD-like pathology [58,60]. CRISPR-mediated amelioration of AD-like pathology in animal models can be achieved by targeting other genes not directly involved in Aβ production, e.g., [61,62]. Overall, the potential applications of CRISPR-Cas9 in the AD field have been successfully tested in a variety of animal models, using different approaches (Table 1). However, more studies are needed to fully assess the presence or absence of off-target genome modification, which may manifest long after the CRISPR-Cas9 technology has been deployed.

## 6. CRISPR-Cas9 in Parkinson’s Disease

PD is a neurodegenerative disorder characterized by the loss of dopaminergic neurons of the basal ganglia and the *substantia nigra*, and the accumulation in these brain regions of toxic deposits called Lewy bodies. Clinically, people affected by this disorder have uncontrollable movements, such as shaking, stiffness, and difficulty with balance and coordination [63]. There are two forms of PD, sporadic and familial, with the latter representing only 10–15% of the cases. The familial forms are caused by mutations in several genes such as SNCA, Parkin, PINK1, DJ-1, and LRRK2. Mutations in SNCA and LRRK2 are responsible for autosomal-dominant PD forms, and mutations in Parkin, PINK1 and DJ-1 are responsible for autosomal recessive forms [64]. While the causes of the sporadic forms are unknown, several environmental and genetic risk factors have been identified. For example, variations in the SNCA and LRRK2 genes increase the risk of developing sporadic PD [65].

Over the last few years, CRISPR-Cas9 technology has been widely employed for PD research to knock out, knock in or modify genes linked to the disease, selectively activate, or repress the expression of key genes, or apport epigenetic modifications. In vitro studies have been mainly used to generate PD cellular models. For example, using induced pluripotent stem cells (iPSCs) from marmosets (a non-human primate), Vermilyea and colleagues utilized CRISPR-Cas9 to introduce the G2019S mutation in the LRRK2 gene. After the iPSCs were differentiated into dopaminergic neurons, they found an increase in ROS production, decreased neuronal viability, and neurite complexity, all features associated with PD [66]. Successful application of CRISPR-Cas9 in generating in vitro models of PD has also been obtained using human embryonic stem cells [67]. CRISPR-Cas9 has also been used to generate in vivo models of PD. For example, Vps35 D620N knock-in mice, which develop some key features of PD, were generated by CRISPR-Cas9-mediated genome engineering [68]. Two independent groups went further and generated non-human primate models of PD [69,70]. Chen and colleagues used a modified CRISPR-Cas9, which allowed them to reduce the off-target modification of the system. Specifically, to target the PINK1 gene, they used a Cas9 gene harboring a D10A mutation, known as nCas9 [69]. As mentioned above, instead of introducing DSBs, mutated nCas9 cuts only one strand of the target DNA, and its frequency of off-target edits is greatly reduced. Indeed, using the nCas9 with a pair of gRNAs targeting the same region of the gene of interest, one can achieve a DSB, while the off-target single-strand breaks are repaired by the base excision repair pathway [7,71]. In an independent study, concomitant knockout of the PINK1 and DJ-1 genes in the *substantia nigra* of monkeys was achieved by a standard CRISPR-Cas9 system delivered by AAV9. This approach led to the development of several key features of PD, including behavioral and neuropathological alterations [70].

To modify the methylation state of the SNCA gene, which is increased in PD patients [72], Guhathakurta and colleagues used a clever CRISPR-dCas9-mediated approach. Specifically, they recruited a specific histone lysine demethylase, JARID1A, to the SNCA promoter of PD-iPSCs. They reported that their system was sufficient to decrease the expression of α-synuclein [73]. This study set the stage for an innovative therapeutic approach for PD.

As in AD, the CRISPR-Cas9 technology in PD has also been used to delete the expression of a mutated gene or directly revert mutations known to cause the disease. A classic example is given by the A53T mutation in the SNCA gene, which is one of the most studied mutations in PD. Rats overexpressing α-synuclein harboring the A53T mutation develop a PD-like phenotype, which can be prevented by CRISPR-Cas9-mediated deletion of the mutated gene [74].

## 7. CRISPR-Cas9 in Huntington’s Disease

Huntington’s disease (HD) is a neurodegenerative disorder caused by a dominant, autosomal mutation in the huntingtin gene (HTT) located on chromosome 4. The mutated gene has extra poly-glutamine (CAG) repeats, and this causes the production of abnormal, toxic proteins [71]. To date, there are no treatments for this disorder, and the molecular mechanisms underlying the disease pathogenesis have yet to be uncovered [75]. Given its mainly genetic causes, HD could be considered the ideal neurodegenerative disease in which to employ CRISPR-Cas9. To this end, different groups, using alternative approaches, have shown that reducing the production of the mutant *htt* allele could be a valid therapeutic approach [76,77,78,79,80].

Like other neurodegenerative diseases, animal models of HD have been proven to be an invaluable tool for studying pathogenic mechanisms associated with the disease. However, these models, which usually are rodents, often do not recapitulate all the key aspects of the disease they are intended to model. Indeed, it has been suggested that to fully recapitulate a neurodegenerative disease, including HD, one needs to use different species other than rodents. Using CRISPR-Cas9, Yan and colleagues introduced 150 CAG repeats into the endogenous pig HTT gene, effectively generating the first successful pig model of HD [81]. Specifically, they targeted exon 1 of the endogenous *htt* gene and used donor DNA containing the 150 CAG repeats and the other homologous regions of the pig *htt* gene. The recombination process was performed in pig fibroblasts, which after proper selection, were transferred by somatic cell nuclear transfer into surrogate pigs to obtain the genetically modified animals [81].

## 8. CRISPR-Cas9 in Amyotrophic Lateral Sclerosis

ALS is a neurodegenerative disorder characterized by the progressive degeneration of motoneurons (MNs) of the motor cortex, brain stem, and spinal cord [82]. The genetic forms of this disease are caused by mutations in the superoxide dismutase 1 (SOD1), TAR DNA Binding Protein-43 (TARDBP), and fused in Sarcoma (FUS) genes, or Chromosome 9 Open Reading Frame 72 (C9ORF72) [82]. Using CRISPR-Cas9 to modify these genes in animal models of ALS and patient-derived induced pluripotent stem cells (iPSCs) can provide important insights into the pathogenic mechanisms underlying ALS [83].

Standard CRISPR-Cas9 has been used to introduce various mutations into the SOD1 gene of iPSCs. The resulting cell lines developed some features associated with the disease [84,85]. Similar experiments have been conducted in vivo with the use of CRISPR-Cas9-mediated homologous recombination to generate multiple C. elegans models, each carrying an independent SOD1 mutation [82]. These animals have been used to dissect different aspects of neurotransmitter alterations associated with ALS [86]. In complementary experiments, and similarly to what has been performed in other neurodegenerative diseases, CRISPR-Cas9 has been used to revert mutations causing diseases in animal models [87,88].

TAR DNA-binding protein 43 (TDP-43) is the major protein that accumulates in ALS. Specifically, the accumulation of its C-terminal fragments appears to track the disease phenotype [89]. To better understand various mechanisms of pathogenesis, multiple groups have successfully employed CRISPR-Cas9 genome editing, paired with specific donor DNA, to introduce human mutations into the endogenous mouse gene encoding TDP-43 [90,91]. These approaches have the major advantages of removing the confounding of having an exogenous promoter drive the expression of TDP-43, such as in the case of the generation of traditional transgenic mouse models.

Complementary CRISPR-Cas9-mediated approaches have been used to correct the C9orf72 repeat expansion. For example, the exonuclease repeat expansion has been replaced with the wildtype version in iPSCs pairing standard CRISPR-Cas9 with a homology-directed repair. This approach led to a complete restoration of the wildtype genotype and phenotype [92]. Recently, a similar approach has been used to correct the C9orf72 alterations in mice [93]. In this case, however, the authors employed two different gRNAs to reduce off-target effects. The results were exciting as the CRISPR-Cas9-mediated correction reversed some key aspects of the ALS-like phenotype caused by mutant C9orf72 [93].

**Table 1:** Examples of the applications of CRISPR/Cas9 in four of the most diffuse neurodegenerative disorders.

Pathology	CRISPER-Cas System	Target	Point Mutation	Organism/ Cell Line	Result	Ref.
**AD**	CRISPR-Cas9	APP gene	3′-UTR of APP	APP-KI mice	reduced Aβ pathology	[49]
			G676R, F681Y, and R684H	mouse	humanized animal models	[50]
			deletion	Tg2576 mice	reduce Aβ production	[54]
			C-terminus			[56]
		Mapt gene	deletion in the transcriptional start codon (exon 1)	Tau knockout mice	C57Bl/6J background resistant to excitotoxicity	[51]
		Plcγ2-P522R variant	P522R	mouse	Plcγ2-P522R knock-in mouse model	[54]
	dCas9	BACE-1	decrease the expression of BACE-1	animal models of AD.	reduction of Aβ production	[58]
	dCas9	ADAM10	increase the expression of ADAM10	animal models of AD.	reduction of Aβ production	[60]
**PD**	CRISPR-Cas9	LRRK2 gene	G2019S	stem cells from marmosets	Modification of features associated with PD	[66]
				embryonic stem cells		[67]
		Vps35	D620N	mouse	Vps35 D620N knock-in (KI) mice	[68]
		PINK	D10A	monkeys	off-target edits reduction	[71]
		PINK1 and DJ-1 genes	deletion		PINK1 and DJ-1 gene knockout model	[70]
	CRISPR-dCas9	SNCA	histone lysine demethylase (JARID1A)	PD-iPSCs	decrease the expression of α-synuclein	[73]
	CRISPR-Cas9		deletion mutated SNCA-A53T	Rats		[74]
**HD**	CRISPR-Cas9	mutant HTT	reducing HTT mutated	In vitro	Modification of features associated with HD	[76,77,78,79,80]
		HTT gene	introduced 150 CAG repeats	pig	pig model of HD	[81]
**ALS**	CRISPR-Cas9	SOD1	independent SOD1 mutation	C. elegans models	animals ASL model	[82]
			several mutations into	iPSCs.	Modification of features associated with ALS	[84,85]
		TDP-43	introduce human mutations	mouse	transgenic mouse models	[90,91]
		C9orf72	correct the C9orf72 repeat with the wildtype gene	iPSCs	restore wild-type genotype and phenotype	[92]
				mice		[93]

## 9. Conclusions

The CRISPR-Cas9 system, with all its variants, has quickly revolutionized gene editing throughout many scientific fields. The ability to edit the genome selectively and accurately has led many to think that medicine was heading towards curing most genetic disorders. However, the many caveats associated with the use of CRISPR-Cas9 to cure neurodegenerative disorders have slowdown its clinical applications, despite the generation of engineered Cas9, such as nCas9, and the use of sophisticated algorithms to design gRNAs, which greatly reduced the probability of off-target edits.

From the discovery science perspective, CRISPR-Cas9 has almost become an essential tool in a scientific laboratory. It is now easily accessible to everyone; for example, the development of a single viral expression vector coding for both Cas9 and the gRNA allows one to modify the genome of an animal model of any neurodegenerative disorder by a simple stereotaxic injection of the viral vector. Furthermore, the use of smaller Cas9 allows the experimenter to pack into the viral vector more control genes (e.g., GFP) or multiple gRNAs.

Flexibility and easy access are also evident in the commercially available Cas9 transgenic mice. Using a backcrossing strategy, one can breed these Cas9 transgenic mice with their favorite animal model of neurodegenerative disorders. After this initial time investment, the experimenter has a tool in which they can modify any gene of interest by a simple injection of a viral vector expressing one or multiple guides. Thus, multiple genes of the same pathways or different pathways may be targeted simultaneously. Overall, the potential of this technique outweighs the potential drawbacks, and as of today, CRISPR-Cas9 technology is the best gene-editing tool available to study neurodegenerative disorders.

## Data Availability

Not applicable.
